# Moderate increase of precipitation stimulates CO_2_ production by regulating soil organic carbon in a saltmarsh

**DOI:** 10.3389/fmicb.2024.1328965

**Published:** 2024-01-24

**Authors:** Lirong Zhang, Guangxuan Han, Lifeng Zhou, Xinge Li, Xiaojie Wang, Xiaoshuai Zhang, Leilei Xiao

**Affiliations:** ^1^CAS Key Laboratory of Coastal Environmental Processes and Ecological Remediation, Yantai Institute of Coastal Zone Research, Chinese Academy of Sciences, Yantai, China; ^2^University of Chinese Academy of Sciences, Beijing, China; ^3^Shandong Key Laboratory of Coastal Environmental Processes, Yantai, China; ^4^The Yellow River Delta Ecological Research Station of Coastal Wetland, Chinese Academy of Sciences, Yantai, China; ^5^School of Geography and Environment, Liaocheng University, Liaocheng, China; ^6^The College of Geography and Environmental Science, Henan University, Kaifeng, China

**Keywords:** precipitation change, saltmarsh, CO_2_ production potential, carbon quantity and quality, microbial r-/K-strategy

## Abstract

Saltmarsh is widely recognized as a blue carbon ecosystem with great carbon storage potential. Yet soil respiration with a major contributor of atmospheric CO_2_ can offset its carbon sink function. Up to date, mechanisms ruling CO_2_ emissions from saltmarsh soil remain unclear. In particular, the effect of precipitation on soil CO_2_ emissions is unclear in coastal wetlands, due the lack of outdoor data in real situations. We conducted a 7-year field manipulation experiment in a saltmarsh in the Yellow River Delta, China. Soil respiration in five treatments (−60%, −40%, +0%, +40%, and + 60% of precipitation) was measured in the field. Topsoils from the last 3 years (2019–2021) were analyzed for CO_2_ production potential by microcosm experiments. Furthermore, quality and quantity of soil organic carbon and microbial function were tested. Results show that only the moderate precipitation rise of +40% induced a 66.2% increase of CO_2_ production potential for the microcosm experiments, whereas other data showed a weak impact. Consistently, soil respiration was also found to be strongest at +40%. The CO_2_ production potential is positively correlated with soil organic carbon, including carbon quantity and quality. But microbial diversity did not show any positive response to precipitation sizes. r-/K-strategy seemed to be a plausible explanation for biological factors. Overall, our finding reveal that a moderate precipitation increase, not decrease or a robust increase, in a saltmarsh is likely to improve soil organic carbon quality and quantity, and bacterial oligotroph:copiotroph ratio, ultimately leading to an enhanced CO_2_ production.

## 1 Introduction

As part of global climate change, precipitation size is constantly changing on a regional or large scale (Alster et al., 2013; IPCC, 2021; Wu et al., 2023). It can alter soil moisture and salinity to influence soil respiration in a variety of ecosystems (Han et al., 2018; Li et al., 2021; Morris et al., 2022). Effects of precipitation events on soil respiration are variable and ecosystem-dependent (Jiang et al., 2013; Su et al., 2023a). For example, strong precipitation is likely to increase soil moisture levels but decrease CO_2_ fluxes in tropical rain forests (Cleveland et al., 2010; Zhang et al., 2015). Similarly, an observation suggested that precipitation events could decrease soil respiration by increasing soil moisture and inducing anoxic conditions in a coastal wetland (Han et al., 2018). In contrast, coastal wetland became a weaker annual CO_2_ absorption due to the extreme precipitation event (Wei et al., 2021). Wang et al. (2019) suggested an active soil respiration to changed precipitation. Increased precipitation stimulated soil respiration by 26.1%, while a reduction by 10.8% responding to low precipitation in a desert steppe (Wang et al., 2019). Further, ambient precipitation determined the sensitivity of soil respiration to different treatments (Li et al., 2023). To data, the response regime and governing factor on soil respiration are still not fully constrained in response to changed precipitation size (Su et al., 2023b), especial for coastal wetland soil sensitive to moisture content (Li et al., 2023).

Increased soil respiration rate by a more large size of precipitation was likely due to favorable soil microbial activity and community composition (Huang et al., 2018) or plant growth (Zhang et al., 2019; Zhou et al., 2019), litter decomposition (Canarini et al., 2017; Qin et al., 2019), carbon substrate availability (Wang et al., 2020), and soil salinization (Han et al., 2015; Zhao et al., 2020). Our previous results revealed that precipitation treatments in a coastal wetland significantly affected soil respiration through a series of abiotic and biotic processes, mainly by changing soil water and salt conditions (Li et al., 2021; Huang et al., 2023). In comparison, some studies showed high levels of precipitation caused soil organic carbon to accumulate, due to oxygen-limitation (Kramer and Chadwick, 2018; Possinger et al., 2020). Moreover, variable responses of dissolved organic carbon to precipitation events were also confirmed (Suseela et al., 2013; Warner et al., 2019). In compared to recalcitrant carbon, the labile one is more preferred by microbes (Chen et al., 2022). To this end, soil carbon quantity and quality reserve more attention to explain the link between precipitation and soil respiration.

Precipitation size can directly affect microbial community and function. Currently, aiming to gain a deeper understanding of interaction of soil organic carbon to microorganisms, ecologists have tried to classify microbial taxa based on their life strategies and resource preferences (e.g., copiotrophs vs. oligotrophs) (Fierer et al., 2007; Cotrufo et al., 2015; Chen et al., 2022), in term of r-/K-strategy. Microbial r-strategies (copiotrophic species) refer to organisms that have rapid growth rate and able to make full use of easily decomposed carbon. In contrast, K-strategy bacteria (oligotrophic species) are characterized by slower growth and have a more efficiency to utilize recalcitrant carbon (Fierer et al., 2007; Ho et al., 2017; Singh, 2018). The r-categories and K-categories provide a useful framework for understanding the traits that functionally similar taxa possess (Fierer et al., 2007; Ho et al., 2017). Precipitation size may affect soil respiration by change of microbial r-/K-strategy.

We hypothesized: (1) The response pattern (positive or negative) of CO_2_ production potential is likely to be distinct across the precipitation gradient; (2) Both quantity and quality of soil organic carbon are involved in the response of soil respiration; (3) Microbial community and r-/K-strategy govern soil respiration. To address the above potential mechanisms of the influence of precipitation change on soil CO_2_ emissions, we conducted a 7-year (2014–2021) field experiment in the Yellow River Delta, China. There were five treatments for this field experiment (−60%, −40%, +0%, +40%, and +60% of precipitation). First, we sampled topsoils in 2019–2021 and conducted some microcosm experiments. A detailed analysis on soil organic carbon characteristics and microbial function was conducted to verify proposed hypothesis.

## 2 Materials and methods

### 2.1 Site description

This study was conducted in the natural marshes (37°45′50″N, 118°59′24″E), which is located in the Yellow River Delta, Shandong, China. The local climate is a warm temperate and continental monsoon climate with a mean annual temperature of 12.9°C and minimum mean temperatures of 26.7°C in July and −2.8°C in January, respectively. The average annual precipitation is 530–630 mm, concentrated mostly in summer (from May to September). The soil texture in this region is sandy clay loam, and the soil type gradually changes from fluvo-aquic soil to saline soil (Han et al., 2018). The main founding species in this place are flood-tolerant *Phragmites australis* and salt-tolerant Suaeda salsa.

### 2.2 Experimental design

The field precipitation experiment, initiated in October 2014, included five treatments: a decrease of 60% (−60%) and 40% (−40%), control, and an increase of 40% (+40%) and 60% (+60%) of precipitation. This experiment was established using a completely randomized block design on the natural vegetation and soil at the Yellow River Delta Ecological Research Station of the Coastal Wetland, Chinese Academy of Science.^1^ In all, 20 plots in total were randomly assigned to the five precipitation treatments, with each treatment being randomly repeated four times. The plot size was 3 × 4 m with 1 m between any two adjacent plots, and a core area of 2 × 3 m was used for measurements. Detailed information is given our previous studies (Li et al., 2021, 2023). Typically, a 21 cm-diameter 8 cm height polyvinyl chloride polymer collar was permanently installed 5 cm into the soil at the center of each plot for the measurement of soil respiration. We measured soil respiration by using an LI-8100 infrared gas analyzer, Li-Cor, Inc., Lincoln, NE, United States, connected to an 8100-103 soil respiration chamber. One or two days before the measurements, all living plants inside the collars were carefully clipped from the soil surface to exclude aboveground plant respiration. To eliminate diurnal variation, the measurements of soil respiration were collected between 8:00 a.m. and 12:00 p.m. (local time).

### 2.3 CO_2_ production potential

For CO_2_ production potential test with microcosm experiment, topsoil samples (0–10 cm) were collected at the end of the 2019–2021 growing seasons (October–November) using a manual stainlesssteel corer of 10 cm diameter. 5 g soils and 5 mL sterile water were added into a anaerobic tube with the total volume of 25 mL. Anaerobic vials were completed with three vacuum/charging cycles of high purity nitrogen, resulting in an anaerobic environment filled with high-purity nitrogen. Then the incubation was conducted at room temperature in a dark environment. The gas was measured and collected every 3 days or so to determine its gas concentration. The incubation was terminated when the gas production concentration peaked or declined. The gas samples were analyzed with a gas chromatograph (GC) (Agilent 7890A, United States) equipped with a flame ionization detector and an automated flow-injection apparatus.

### 2.4 Soil property analyses

Based on the analysis of CO_2_ production potential, the samples obtained in 2021 were used to further soil property and microbial analysis. Total carbon (TC) and total nitrogen (TN) were measured on an elemental analyzer (Vario MACRO cube, Elementar Analysensysteme, Germany). We removed total inorganic carbon with hydrochloric acid (HCl, 1 mol L^−1^) from the samples, and then measured total organic carbon (TOC) on the elemental analyzer. Soil carbon (C) quality was characterized by relative peak area of aliphatic and aromatic hydrocarbons, which was determined using Fourier transform infrared spectroscopy (FTIR, Nicolet iS5, Thermo Scientific). Before the determination, the air-dried soil was screened with a 300-mesh screen for small particles, and ground with an agate mortar and pestle for further homogenization. The reflection spectra of 400 ~ 4,000 cm^−1^ were obtained by FTIR, and the relative peak areas of different characteristic peaks were calculated. The abundance of other organic carbon functional groups can be represented by the relative peak area of corresponding characteristic peaks. In this paper, soil carbon quality can be represented by the ratio of aliphatic groups to aromatics (rA2930:rA1635 ratio), and the higher the ratio, the better the quality (Chen et al., 2022). For example, the ratio of rA2930:rA1635 is a proxy of the ratio of labile carbon (aliphatic) and not easily decomposed carbon (aromatics). Because aliphatic compounds are usually composed of straight, branched, or annular carbon atoms connected by single or multiple carbon–carbon bonds. In contrast, aromatic compounds are composed of benzene rings and their derivatives with a stable ring structure and high saturation. Therefore, aliphatic reactions are likely to be more active than aromatic reactions.

### 2.5 Molecular analyses of microbial community composition

We used a PowerSoil DNA Kit (Qiagen) to extract DNA. As is widely acknowledged, V4 region of the 16S rRNA gene with the primers 515F (5′-GTGYCAGCMGCCGCGGTAA-3′) and 806R (5′-GGACTACNVGGGTWTCTAAT-3′) can be used to analyze bacterial community. Similarly, the primers ITS1-F (5′-CTTGGTCATTTAGAGGAAGTAA-3′) and ITS2 (5′-GCTGCGTTCTTCATCGATGC-3′) were used to test fungal community by amplifying the internal transcribed spacer (ITS1) region. To well distinguish the samples after sequencing, barcode sequences were added to the primers. The PCR conditions for both the 16S and ITS amplification procedures can refer to Chen et al. (2022). The Illumina HiSeq platform with a 150 bp paired-end sequencing kit was used to complete amplicons sequence. K-strategy of bacterial communities are in general with fewer rrn copies, whereas those with more rrn copies are classified to a r-strategy (Roller et al., 2016). CO_2_ production potential under precipitation treatments might intimately rely on microbial community or the activity of certain specific microorganisms to in anaerobic environments. Therefore, metabolically active bacteria and fungal were both tested in our experiments. Only the genes with an abundance at top 10 were presented. Sequence data associated with this project have been deposited in the NCBI Short Read Archive database (Accession Number: PRJNA1060787).

### 2.6 Statistical analysis

Data were presented as the mean ± standard deviation of triplicate cultures. All statistical analyses were performed using Origin 2021 (Origin Lab Corporation, United States) software. A *t*-test was used to analyze the significance level. Significance was accepted at the *p* < 0.05 level of probability.

## 3 Results and discussion

Here, we conducted in field experiments to simulate −60, −40%, +40%, and +60% precipitation vs. ambient conditions from 2014 in the Yellow River Delta region of China. Soil respiration from a 7-year of simulation of precipitation in 2021 was tested. The following sections present results on soil CO_2_ production. Further, the physical and chemical properties of soils, the quantity and quality of organic carbon, and the role of microorganisms on CO_2_ production were discussed.

### 3.1 Soil CO_2_ production

**Figure 1:**
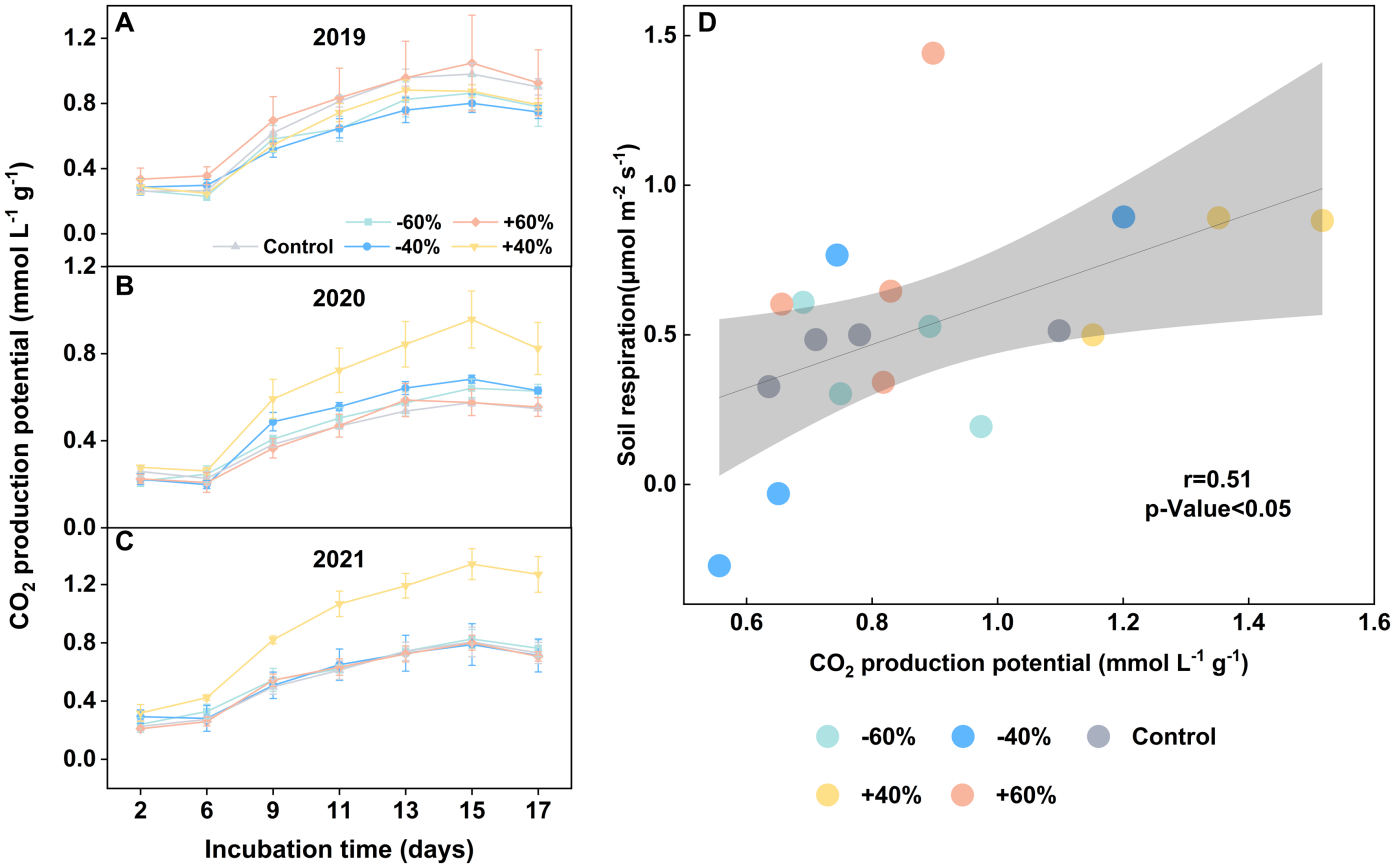
Variation of CO_2_ production potential in topsoil under precipitation treatments in 2019 ~ 2021 **(A–C)**. CO_2_ correlation analysis of *in situ* soil respiration and laboratory incubation for samples from 2021 **(D)**. Vertical bars indicate standard errors. “−60%” and “−40%”: 60 and 40% decreases in precipitation, respectively; “Control”: ambient precipitation; “+40%” and “+60%”: 40 and 60% increases in precipitation, respectively. Note: three replicates were used in this study in “+40%” group.

There was no significant effect on CO_2_ production from precipitation sizes in 2019 (Figure 1A), possible due to an extreme rainfall this year (Li et al., 2023). In two consecutive years (2021 and 2022), however, we clearly observed that a + 40% increase of precipitation boosted CO_2_ production (Figures 1B,C). This stimulated influence was much stronger than that with +60%. Specially, we found a good match between soil respiration from *in situ* experiment (Li et al., 2023) and the microcosm experiment (Figure 1D). Therefore, we confirmed that a moderate increase of precipitation, around +40%, can stimulate a peak of soil mineralization. Furthermore, the effect of decreased precipitations on soil respiration is less obvious. Our results also further suggested an asymmetric response of soil respiration to precipitation sizes in coastal wetland (Li et al., 2021), same as some terrestrial ecosystems (Du et al., 2020; Li et al., 2020).

Precipitation treatment can change the physical and chemical properties of soil. Based on the FTIR spectra data (relative peak area) of the soils, the mineral composition is mainly organosilicon (Supplementary Table S1), and precipitation treatment had no obvious effect on CO_2_ production (*p* > 0.05). We found that a moderate increase in precipitation (+40%) decreased soil conductivity (Table 1). And similar results also have been found in some multi-year field manipulation experiments (Han et al., 2021; Huang et al., 2023). However, no relation between soil electric conductivity and CO_2_ production was discovered (*p* > 0.05). Moderate increase of precipitation (+40%) can lead to the increase of total nitrogen, total carbon and total plant biomass (Table 1). Precipitation may regulate maximum plant biomass accumulation through water and salt transport (Chu et al., 2021; Li et al., 2023). We also found that soil carbon and nitrogen significantly responded to precipitation size and affected soil respiration.

**Table 1:** Basic physicochemistry of soils and plant for five treatments.

Treatment	SM (%)	ST (°C)	Soil EC (mS cm^−1^)	TC (g kg^−1^)	TN (g kg^−1^)	TB (g m^−2^)
−60%	33.55a	14.66a	4.31a	18.18a	0.89a	1818.52a
−40%	32.06a	14.12ab	3.71a	17.71a	0.85a	1924.33ab
Control	34.24ab	13.49bc	3.38a	18.20a	0.92a	2116.42b
+40%	36.75bc	13.10c	1.16b	22.13b	1.27b	2587.72c
+60%	38.59c	13.00c	1.73b	17.71a	0.86a	2855.97c

SM, Soil moisture; ST, soil temperature; soil EC, soil electric conductivity; TPB, total plant biomass; TC, total carbon; TN, total nitrogen; “−60%” and “−40%”: 60 and 40% decreases in precipitation, respectively; “Control”: ambient precipitation; “+40%” and “+60%”: 40 and 60% increases in precipitation, respectively.

### 3.2 Organic carbon quality and quantity

**Figure 2:**
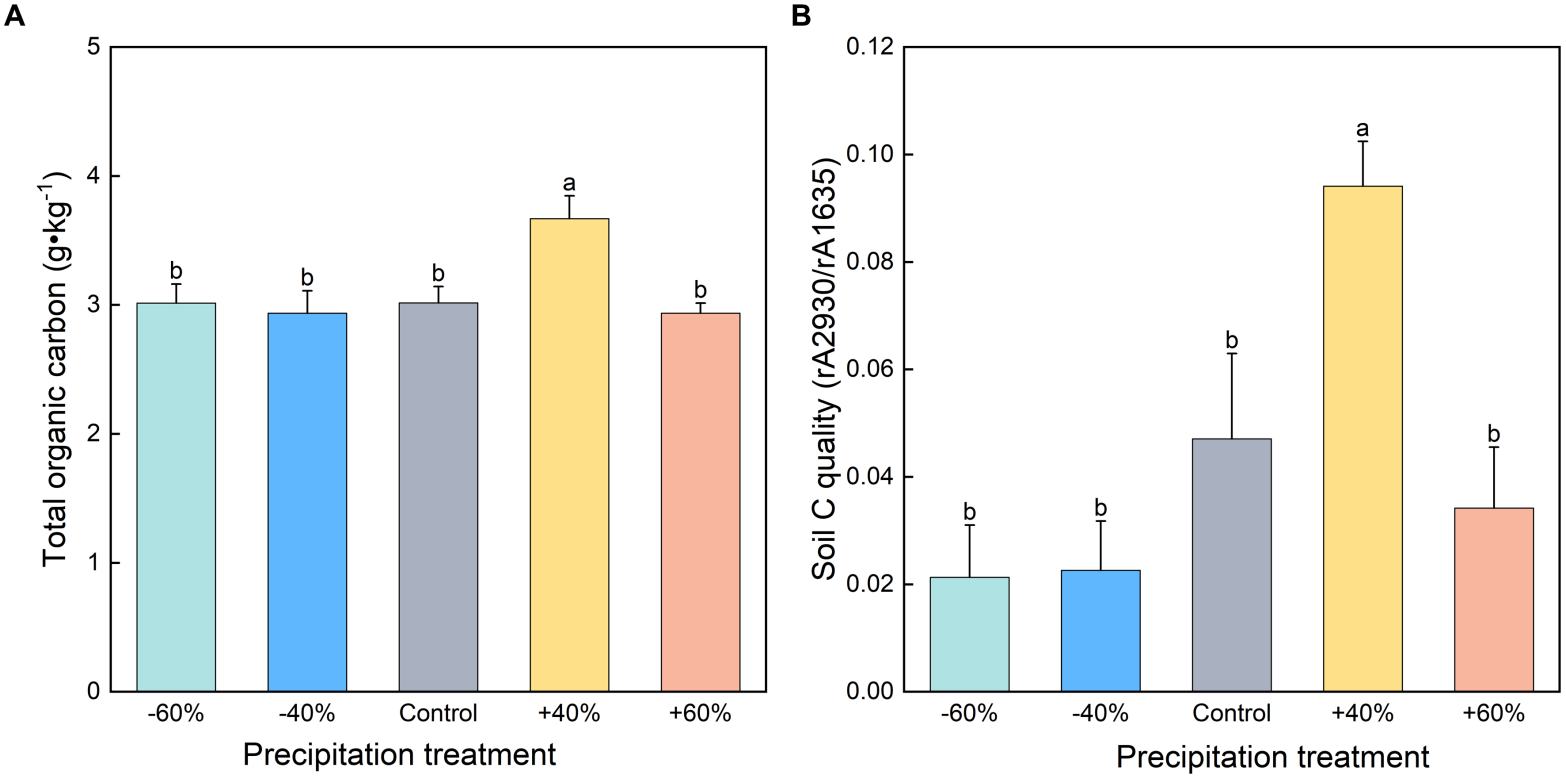
Soil total organic carbon **(A)** and carbon quality **(B)** of topsoil under different precipitation treatments. Different small letters indicate significant differences between precipitation treatments at *p* < 0. 05.

Our study illustrated that a moderate increase in precipitation increased TOC content by +107.6% (Figure 2A). By contrast, the −60%, −40%, and +60% treatments had no significant difference. A positive correlation between CO_2_ production potential and total organic carbon content was discovered (Supplementary Figure S1A). It was suggested that increased precipitation can increase SOC stocks by reducing soil salinity (Ding et al., 2015; Sun et al., 2023). Moreover, the precipitation effect can be directly associated with an increase of net primary production of ecosystems, thus increasing organic matter deposition in soil (Singh, 2018). Carbon content of plants was improved by high precipitation (Table 1), however, we did not find a direct correlation between plant carbon content and soil respiration (*p* > 0.05). High soil carbon content may be regarded as a strong sign for soil respiration (Abdalla et al., 2021; Chen et al., 2023). In addition to carbon quantity, we also found that its quality also governed soil respiration (Supplementary Figure S1B). For example, treatment by +40% precipitation significantly enhanced carbon quality by 99.8% in comparison with the control (Figure 2B). Soil organic carbon and labile carbon along a precipitation gradient was also revealed along the Northeast China transect (Wang et al., 2005) and Qinghai-Tibet Plateau (Wang et al., 2022). It is reasonable that the high carbon quality facilitates soil respiration (Li et al., 2023a). In this study, we cannot rule out the effect of total nitrogen on soil respiration (Wang et al., 2019). Because a significant high of total nitrogen was found in the treatment with +40% precipitation. Therefore, as two important sources of microbial growth, carbon and nitrogen, both respond to changes in precipitation, further affecting soil respiration and CO_2_ emissions. For the detailed carbon components, we did not find any difference for alcohol phenols and polysaccharides (Supplementary Table S1), suggesting that these components had weak response to precipitation size.

### 3.3 Bacterial r-/K-strategy related to CO_2_ production

**Figure 3:**
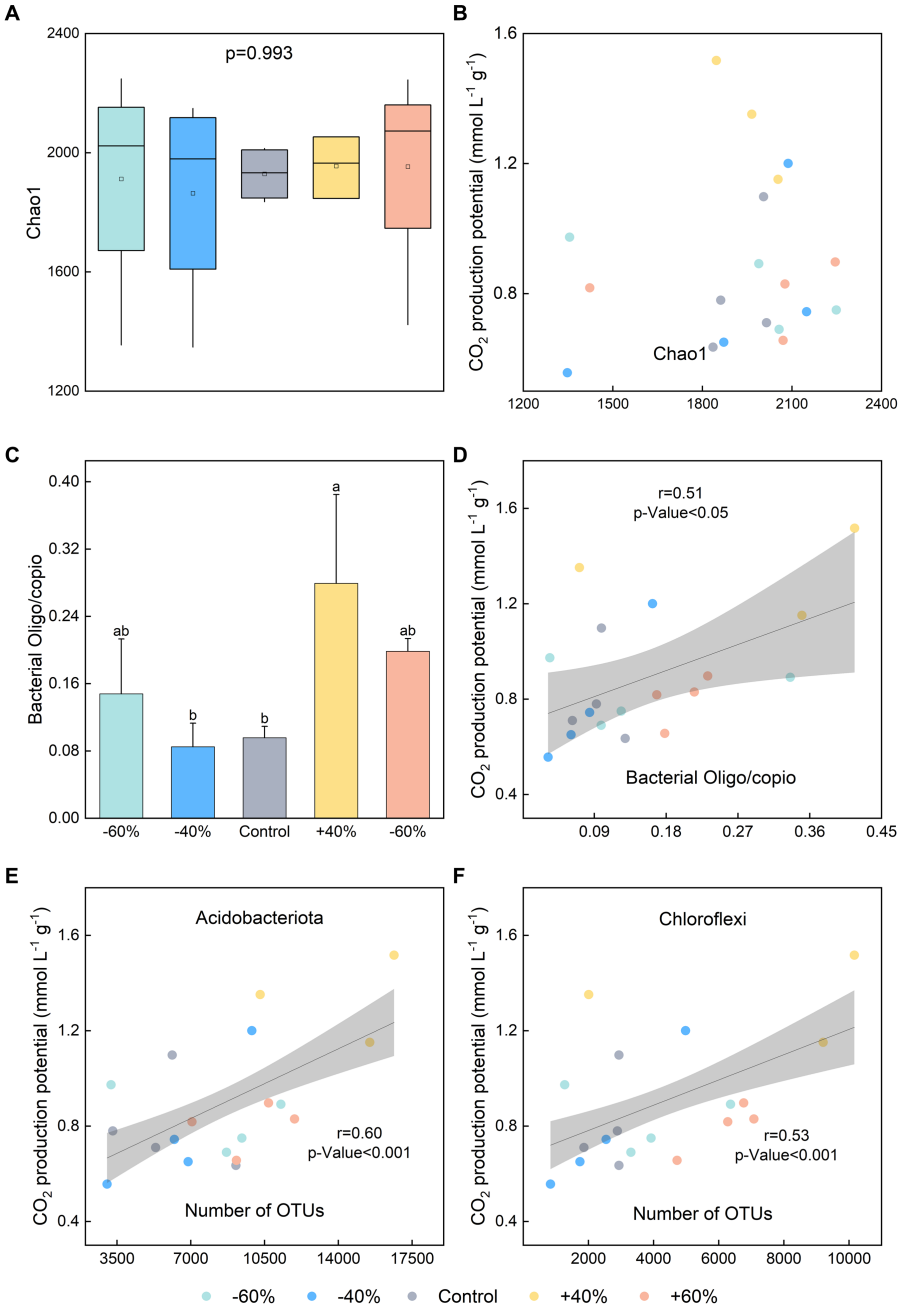
Effects of different precipitation sizes on soil microbial functions and specific species. **(A)** Soil bacterial diversity. **(B)** Correlation between soil bacterial diversity and CO_2_ production potential. **(C)** Oligotroph:copiotroph ratio of soil bacterial communities. **(D)** Correlation between oligotroph:copiotroph ratio of soil bacterial communities and CO_2_ production potential. **(E)** Correlation between the abundance of Actinobacteriota (Actinobacteria phylum) and CO_2_ concentration in the topsoil. **(F)** Correlation between the abundance of Chloroflexi (Chloroflexi phylum) and CO_2_ concentration. Different letters indicate significant differences between treatments, *p* < 0.05.

Out of our expectation, the effect of precipitation on CO_2_ production potential did not depend on microbial diversity (Figures 3A,B, Supplementary Figure S2). In contrast, bacterial r-/K-selected categories were likely to explain microbial response to precipitation sizes (Figures 3C,D). It was demonstrated that bacterial communities in C-rich soils were dominated by K-strategists, while those in the comparatively C-poor soils are dominated by r-strategists (Fierer et al., 2007; Chen et al., 2022). For other extreme conditions, however, K-strategist microbes were also found in carbon-rich environments (Angel et al., 2010; Cary et al., 2010). In this study, oxygen restriction appears to be a factor affecting r-/K- strategies. Oligotrophic bacteria, such as Actinobacteriota and Chloroflexi, were likely to be survive in harsh environments than polytrophic bacteria under anaerobic conditions. Specially, Actinobacteriota and Chloroflexi microorganisms were positively correlated with CO_2_ production potential (Figures 3E,F). Oligotroph:copiotroph ratio was also able to use as a characterization and indication of CO_2_ production potential. With an obvious difference, fungal r/K-selected category was not likely to govern CO_2_ production (Supplementary Figure S3). Thus, bacteria may be more tolerant of anaerobic environments and actively response to precipitation size change, which will result in corresponding change of CO_2_ production.

Taken together, precipitation sizes can affect soil organic carbon quality and quantity and microbial r-/K-strategies. For example, a moderate precipitation size, not a decrease or robust increase, can stimulate the strongest CO_2_ emission. The change of soil carbon resource is likely to regulate bacterial r-/K-strategies. Finally, soil respiration and CO_2_ production can show different scenario in response to precipitation size. Thus, this work proposes precipitation size affects soil microorganisms through biotic and abiotic factors, ultimately leading to differences in soil respiration and CO_2_ emissions.

## Data availability statement

The 16S rRNA and ITS sequencing data have been deposited in the National Center for Biotechnology Information (NCBI) Short Read Archive database (SRA, https://www.ncbi.nlm.nih.gov/bioproject/PRJNA1060787).

## Author contributions

LZha: Data curation, Formal analysis, Investigation, Methodology, Software, Visualization, Writing – original draft, Writing – review & editing. GH: Conceptualization, Data curation, Investigation, Supervision, Writing – original draft, Writing – review & editing. LZho: Data curation, Investigation, Writing – review & editing. XL: Data curation, Investigation, Writing – review & editing. XW: Supervision, Writing – review & editing. XZ: Supervision, Writing – review & editing. LX: Conceptualization, Data curation, Investigation, Supervision, Visualization, Writing – original draft, Writing – review & editing.

## Footnotes

^1^ http://hhm.cern.ac.cn/
