# Supplementary material for: Moderate increase of precipitation stimulates CO2 production by regulating soil organic carbon in a saltmarsh
Source: Front Microbiol. 2024 Jan 24;15:1328965. doi: 10.3389/fmicb.2024.1328965 (PMC10847529; doi:10.3389/fmicb.2024.1328965)
Supplement: Supplementary file 1 [file Data_Sheet_1.docx]

Supplementary information for

**Moderate increase of precipitation stimulates CO_2_ production by regulating soil organic carbon in a marsh**

Lirong Zhang^1,3^, Guangxuan Han^1,2,4^, Lifeng Zhou^1,5^, Xinge Li^1,3^, Xiaojie Wang^1,2,4^, Xiaoshuai Zhang^1,2,4^, Leilei Xiao^1,2,4*^

Table.S1 FTIR spectra data (relative peak area) of the soils.

| Treatment | Alcohol phenols  3623  cm^-1^ | | Polysaccharides  3416  cm^-1^ | Aliphatic  2930  cm^-1^ | Aromatic  1635  cm^-1^ | | Carbohydrate  1030  cm^-1^ | Minerals  (organosilicon)  777  cm^-1^ |
| --- | --- | --- | --- | --- | --- | --- | --- | --- |
| -60% | 3.570a | 8.485a | | 0.026a | 1.384a | 76.342a | | 10.193a |
| -40% | 3.275a | 8.160a | | 0.031ab | 1.575a | 78.306a | | 8.653a |
| Control | 3.202a | 8.257a | | 0.084bc | 1.450a | 79.443a | | 7.564a |
| +40% | 2.716a | 6.004a | | 0.132c | 1.430a | 82.873a | | 6.844a |
| +60% | 3.480a | 8.846a | | 0.053ab | 1.462a | 77.635a | | 8.524a |

Note: Different lowercase letters indicate significant differences between precipitation treatments at *p* < 0. 05.


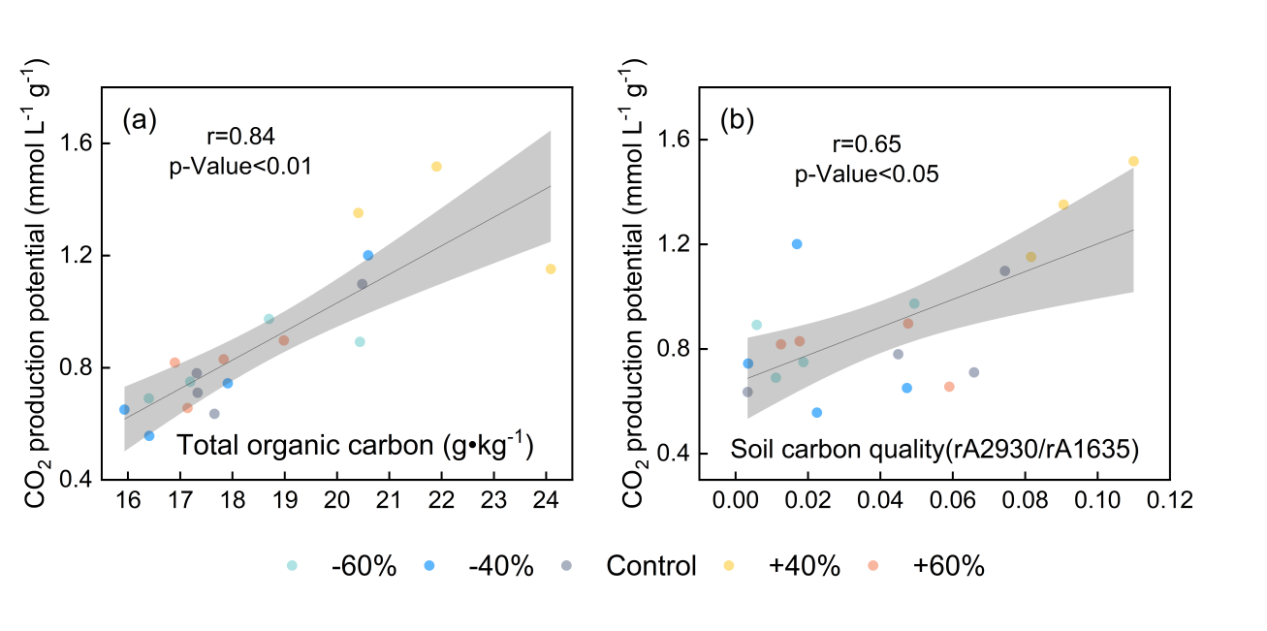


Figure S1. Soil total organic carbon (a) and C quality (b) of topsoil correlation with CO_2_ production potential.


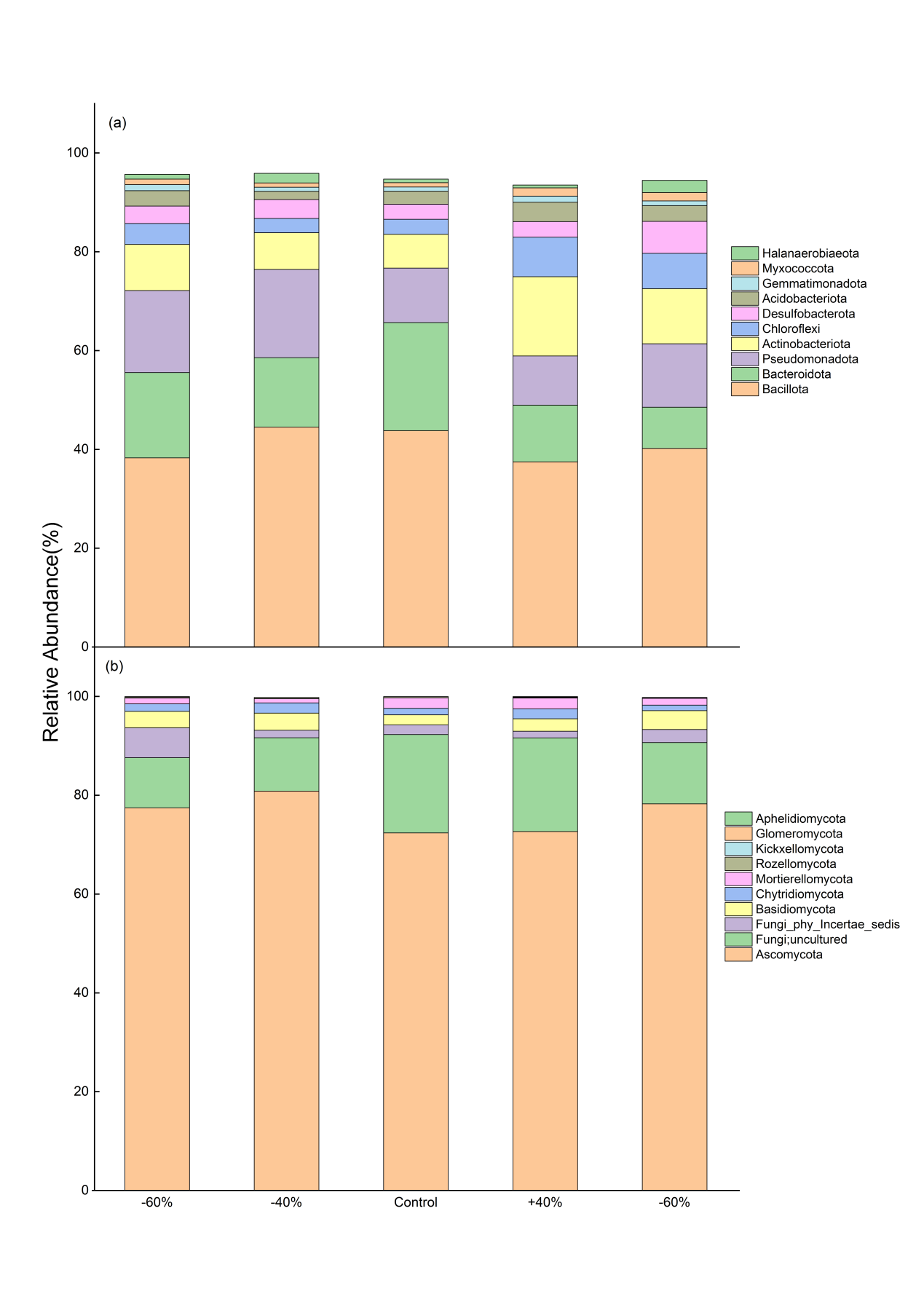


Figure S2. Relative abundances of different bacterial and fungal phyla. (a) Bacterial community composition. (b) Fungal community composition.


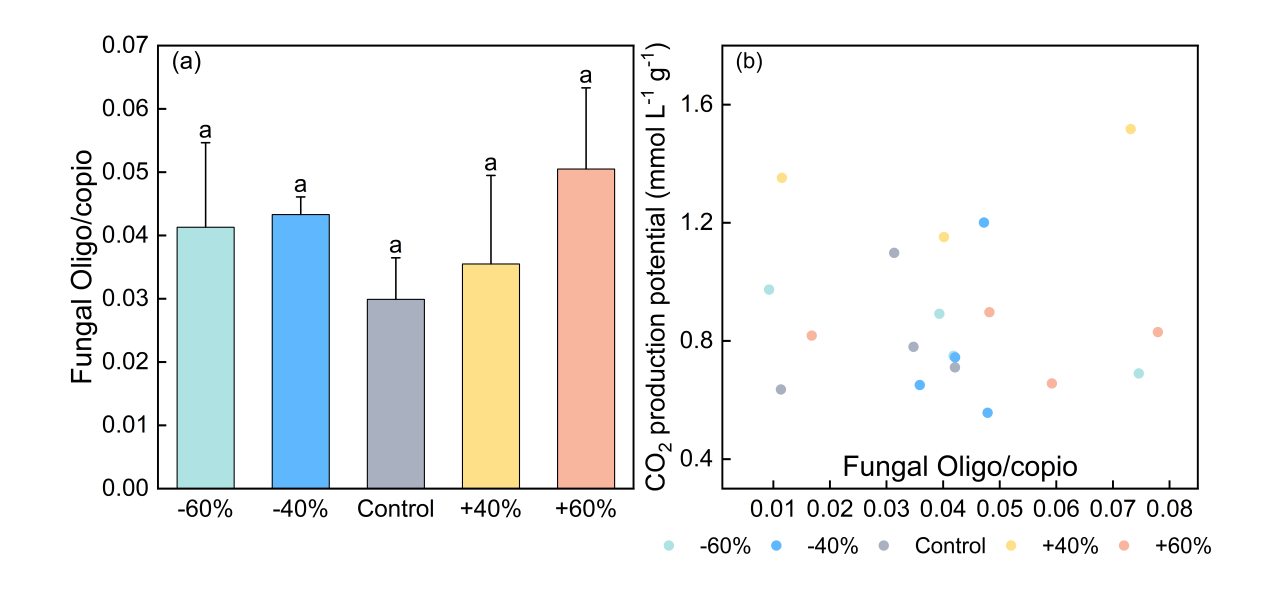


Figure S3. Effects of different precipitation treatments on the r-/K-strategy of soil fungi. (a) Oligotrophic:commensal proportions(OCP) of soil fungal communities. (b) Correlation between oligotrophic:commensal proportions of soil fungal communities and CO2 production potential.
